# Emergence and migration of trunk neural crest cells in a snake, the California Kingsnake (*Lampropeltis getula californiae*)

**DOI:** 10.1186/1471-213X-10-52

**Published:** 2010-05-18

**Authors:** Michelle Reyes, Katrina Zandberg, Iska Desmawati, Maria E de Bellard

**Affiliations:** 1 California State University Northridge, Biology Dept., MC 8303. 18111 Nordhoff Street., Northridge, CA 91330, USA; 2 Institute Technology Sepuluh Nopember, Biology Department, Faculty of Mathematics and Natural Science, Surabaya, Indonesia

## Abstract

**Background:**

The neural crest is a group of multipotent cells that emerges after an epithelial-to-mesenchymal transition from the dorsal neural tube early during development. These cells then migrate throughout the embryo, giving rise to a wide variety derivatives including the peripheral nervous system, craniofacial skeleton, pigment cells, and endocrine organs. While much is known about neural crest cells in mammals, birds, amphibians and fish, relatively little is known about their development in non-avian reptiles like snakes and lizards.

**Results:**

In this study, we show for the first time ever trunk neural crest migration in a snake by labeling it with DiI and immunofluorescence. As in birds and mammals, we find that early migrating trunk neural crest cells use both a ventromedial pathway and an inter-somitic pathway in the snake. However, unlike birds and mammals, we also observed large numbers of late migrating neural crest cells utilizing the inter-somitic pathway in snake.

**Conclusions:**

We found that while trunk neural crest migration in snakes is very similar to that of other amniotes, the inter-somitic pathway is used more extensively by late-migrating trunk neural crest cells in snake.

## Background

The neural crest is a group of multipotent cells that emerge after an epithelial-to-mesenchymal transition from the dorsal neural tube early after neural tube closure. These cells give rise to a wide variety of neuronal and glial derivatives in the peripheral nervous system, as well as parts of the head skeleton and endocrine organs [1,2]. In jawed, anamniote vertebrates like sharks and teleosts, neural crest cells also give rise to electrosensory organs [3] and fin mesenchyme [4]. The neural crest in the trunk portion of an embryo has been found to follow different migratory pathways in different organisms. In amniotes trunk neural crest cells will follow two main courses: a ventromedial pathway through the rostral part of somites, and a dorsolateral pathway between somites and ectoderm [5]. In amphibians, trunk neural crest follows a dorsal pathway into the fin and a ventral pathway between the neural tube and the caudal portion of the somite [6]. In zebrafish, trunk neural crest predominantly migrates between the neural tube and somites as in amphibians [7,8].

The origin of the neural crest was an important event in vertebrate given that it forms most of the craniofacial skeleton [9]. Agnathans (like lampreys) [10,11], teleosts (bony fish) and amphibians clearly possess identifiable cranial neural crest streams that are similar to those observed in amniotes [8,12]. However, the trunk neural crest is less prominent in anamniotes [13,14], which appear to have fewer trunk neural crest cells than amniotes [15-17].

**Figure 1:**
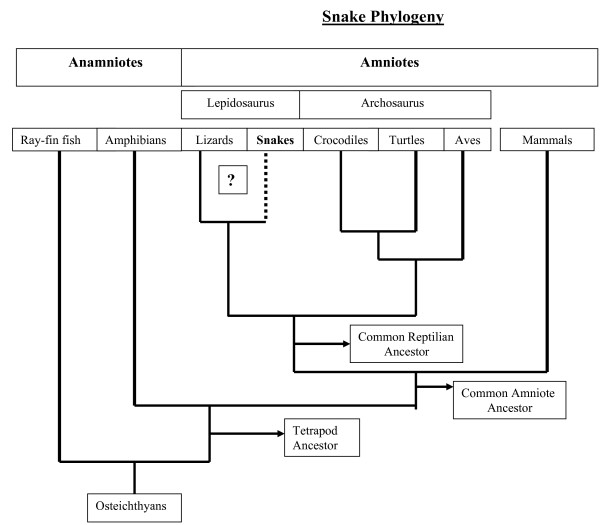
**Phylogeny of Snake**. We describe the phylogenetic position of snakes and other reptilians according to the most recent consensus [18]. The box with question marks refers to organisms for which neural crest migration has not been reported. The figure was drawn by the author, based upon Shedlock's (2007) recent phylogenetic analysis

Recent molecular phylogenies have placed lepidosaurs (tuataras, snakes and lizards) as the most basal non-mammalian (reptilian) amniotes [18]. This contradicts the classical view of anapsids (turtles) as basal reptiles, [19,20] (Fig. 1), instead grouping them with archosaurs (birds and crocodilians). The affinity of turtles and archosaurs has also been supported by recent work showing that neural crest migration in turtles and alligators is very similar to that of birds [21-24]. In either case, lepidosaurs are a critical group for comparisons between amniotes, as they represent one of the three extant groups' non-mammalian amniotes.

Among lepidosaurs, snakes provide an especially interesting case for evolutionary developmental studies as they display a mix of basal and derived amniote features. For example, the development of somites in snakes goes through a similar pattern as in other amniotes, albeit more rapid [25] generating in excess of 300 somites. It is unknown how the basal and derived features of snakes are reflected in the neural crest as neural crest development and migration is poorly described in lepidosaurs.

To better understand the evolution of neural crest migratory patterning in amniotes, we examined the neural crest in the trunk of snake embryos using vital dye labeling and fluorescent immunohistochemistry. We found that trunk neural crest migration in snakes closely follows the patterns observed in turtle, birds, and mammals, with most early-migrating trunk neural crest cells traveling through the anterior portion of the somites and a few migrating between somites. However, we also observed a large number of late-migrating trunk neural crest cells moving between the somites in snake, suggesting the prominence of this pathway may have been accentuated in snakes or reduced in other amniotes. Finally, we observed differences in the structure of a major trunk neural crest derivative, the dorsal root ganglia (DRGs) between non-avian reptiles, birds and mammals.

## Results

### DiI labeling of neural crest

**Figure 2:**
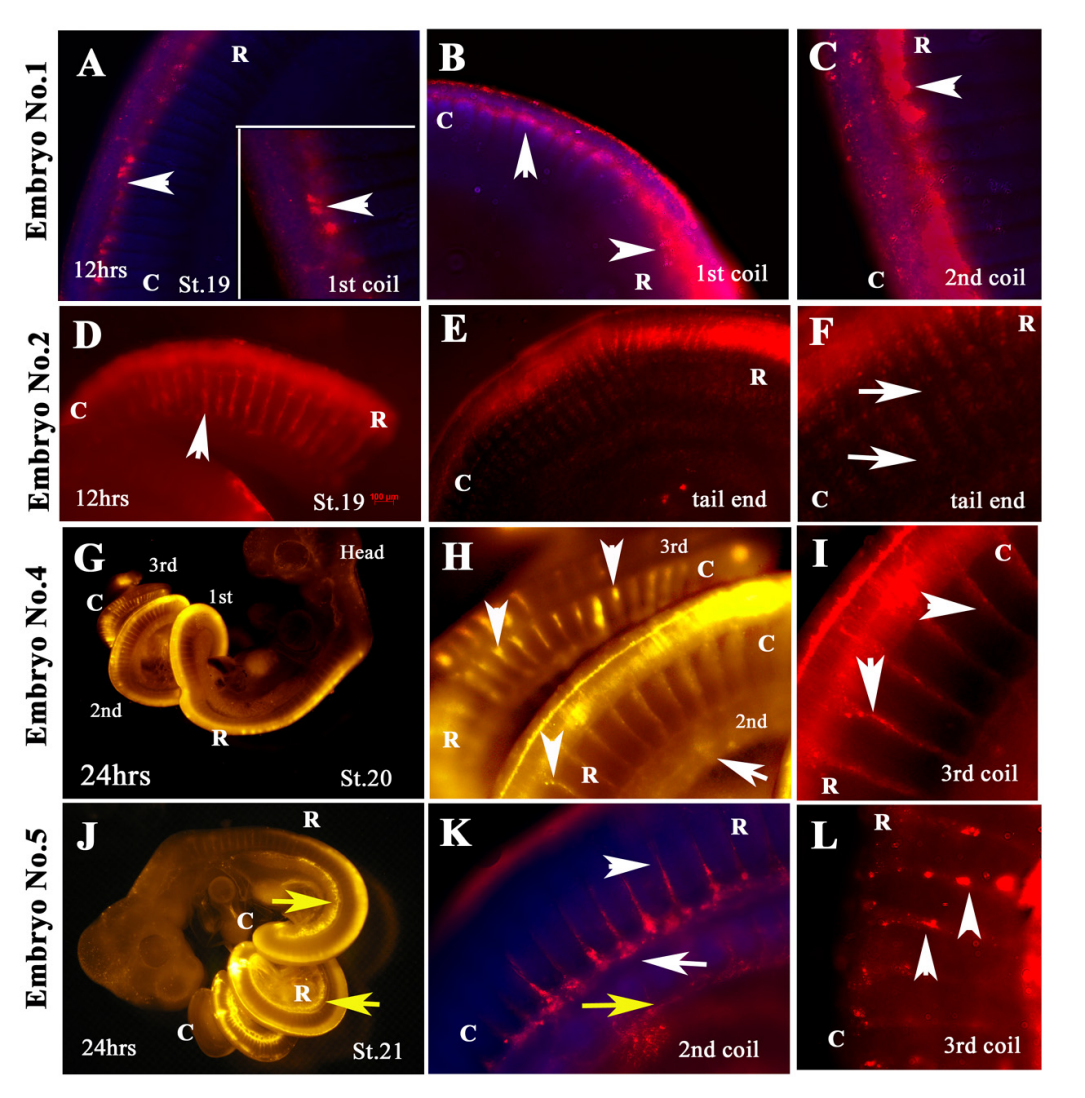
**Snake DiI injection**. Snake embryos (1,2,4 and 5) were removed from the egg and the neural tube injected with DiI. After 12 hrs (**A-F**) or 24 hrs (**G-L**) of incubation embryos were fixed and photographed for migrating DiI-positive neural crest cells. Each embryo is shown in sequential higher magnifications. **Embryo 1 **show delaminated neural crest cells (arrowheads **A-C**), although it did not have substantial number of crest cells migrating in streams (higher magnification in panels **B, C**). **Embryo 2 **had clear robust migrating neural crest cells in streams (arrowhead in **D **for first coil). At the tail level the cells migrated on the rostral portion of the somites, further magnified in panel F. **Embryos 4 and 5 **showed a wider range of DiI labeling since it was incubated for 24 hrs and DiI labeling reached the tail end (**G-L**). **Embryo 4 **neural crest cells migrated as well as streams on the rostral side of somites (arrowheads in H and I) both in 2^nd ^and 3^rd ^coils as well as into the mesonephroi (arrows in **H**). **Embryo 5 **showed the rostrally migrating cells, in the 2^nd ^coil we observe that cells were migrating between the somites (arrowhead in **K**) as well as cells along what is the presumptive aorta (arrow in **K**), also as for Embryo 4, DiI cells reached the mesonephroi (yellow arrows in **J**, **K**). **L **showed higher magnification of 3^rd ^more caudal coil with individual cells migrating on the rostral portion of the somites (arrowheads in **L**). **R **is for rostral; **C **is for caudal orientation in the embryo coils.

We tested two strategies to guarantee that embryos will survive after labeling with DiI. A total of 2/4 embryos cultured with the first strategy at either 37°C or 25°C in a combination of DMEM/FBS and snake yolk, survived after 12 hrs of incubation, by the time of fixation (Additional file 1 and Fig. 2). The second strategy (keeping the embryos in DMEM/FBS plus much of their surrounding membranes intact) worked much better; in this case all embryos were alive up to 24 hrs later (Fig. 2). Of this second batch 2/4 embryos survived and presented a thoroughly DiI-labeled neural tube and migrated neural crest cells along its rostro-caudal axis. All the embryos were injected either on the second or third day after oviposition and were at approximately st.19-20 [26]. However, we found difficult to correlate the development of cranial features in our *Lampropeltis *with the *Thamnophis *used by Zehr to accurately define stages of our embryos. See Additional file 1 for more details on each embryo.

Neural crest migration in vertebrate embryos progresses in a rostro-caudal manner, in other words, at a given stage of development, the cells emigrating caudally are the early-migrating cells while the cells emigrating rostrally correspond to later-emigrating cells. The first observation from the DiI labeled embryos was that snake neural crest migration followed this same pattern. At the caudal level, where neural crest cells are starting to emerge from the dorsal neural tube, neural crest cells were migrating as streams of cells avoiding the caudal third of the somite (Fig. 2E-F, H-I and 2L). At more rostral levels, usually by the 2^nd ^coil, neural crest cells migrated as a small group of cells on what seemed to be the most rostral portion of the somites and the inter-somitic space (Fig. 2D, H-I, K, L).

Embryo No.1 had abundant delaminated DiI-labeled cells, although these cells did not enter the ventromedial pathway (Fig. 2A-C). Sections of these embryo showed that very few cells migrated into regions usually populated by neural crest cells, i.e. dorsal aorta, mesonephroi, sensory ganglia, heart (data not shown). Embryo No.2 presented extensive delaminated cells along the rostral somites, especially at the tail level (Fig. 2E, F), while at the level of first coil the migrated cells on the rostral third of the somites (Fig. 2D). Embryo No.4 showed the best labeling of all (Fig. 2G-I) followed by Embryo No.5. Both embryos showed extensive streams of cells along the rostro-caudal axis on the dorsal portion of the somites and by the mesonephroi (Fig. 2H, K). Higher power pictures showed that these DiI streams had punctuated labeling, suggestive of individual migrating cells (Fig. 2I, L).

**Figure 3:**
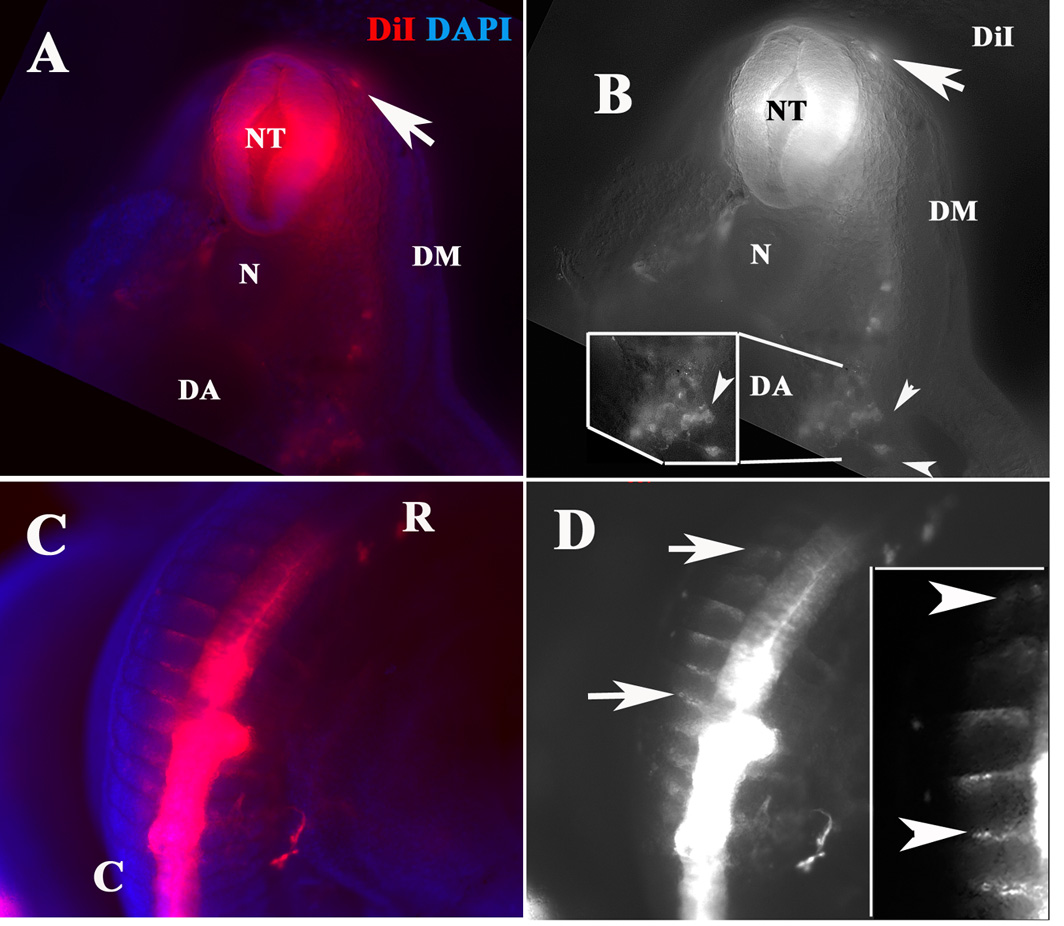
**Migrating cells travel same pathways as neural crest cells**. **A **and **B**: Thick section of a DiI (red) and DAPI (blue) labeled snake embryo (Embryo 2 incubated for 12 hrs) on the second coil showed cells that migrated along the ventromedial pathway and settled by the dorsal aorta (**D, A**). Insert in **B **shows cells in more detail by the aorta. There was also a single DiI cell migrating sub-ectodermally (arrow in **A, B**). **C **and **D **show DiI (red) and DAPI (blue) labeled cells migrating on the most rostral portion of the somite (arrows in **D **point to area of the insert in **D **at higher magnification). This embryo (3 days POP) was labeled with DiI and incubated for 12 hrs at 37°C. **N: **notochord, **DM: **dermomyotome, **NT: **neural tube. **R **is for rostral; **C **is for caudal orientation in the embryo coils.

Thick sections through Embryo 2 (12 hrs incubation) showed that the rostral migratory stream in snakes follows a ventromedial pathway similar to that of birds and mammals (Fig. 3A). Delaminated neural crest cells were distributed in classic regions: ventromedial path between the neural tube and dermomyotome, circumventing the notochord and reaching the dorsal aorta region where they gathered in large numbers (Fig. 3B). A dorsal view of this Embryo 2 highlighted the rostral streams of DiI labeled cells (Fig. 3C, D) while insert shows a higher magnification where individual migrating cells (not motor axons) can be distinguished.

**Figure 4:**
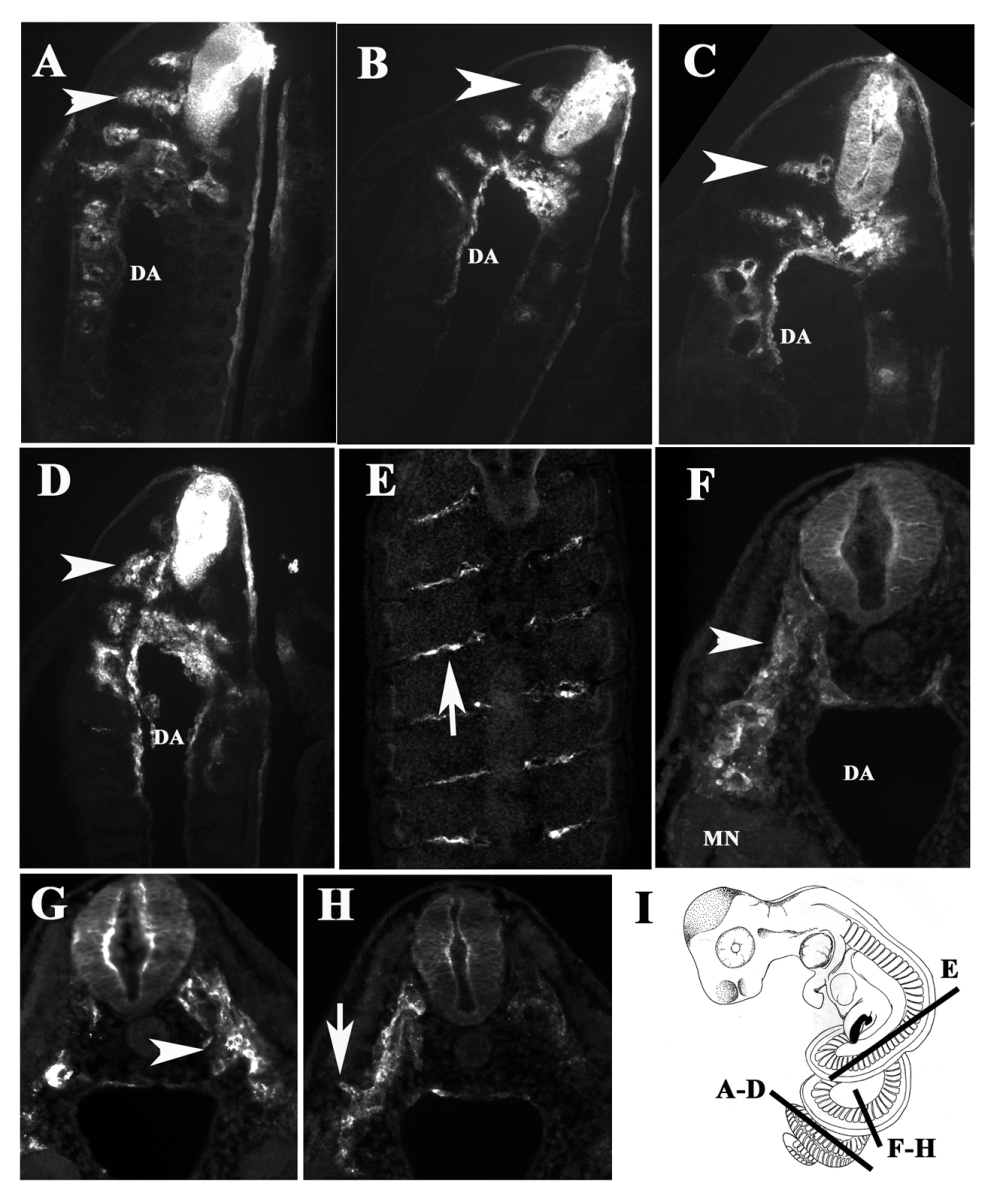
**Sections through DiI labeled snake embryo**. Snake embryos (**Embryo 3 **and **4**) were cryo-sectioned. **A-E **shows sections starting at the most caudal portion and ending at the most rostral portion of st.20 **Embryo 3**. Neural crest cells showed robust migration in the tail region (arrowheads in **A-C**) and at more rostral levels the numbers were fewer and cells were found along the inter-somitic space (arrow in **E**). **F-H **show a transverse section close to the tail, showing thick streams of neural crest migrating ventromedially (**F, G**) or distally as if beginning to enter the sub-ectodermal space (arrow in **H**). **I **Cartoon depicting the axial location and orientation of these sections. **DA**: dorsal aorta. **MN**: mesonephroi. **R **is for rostral; **C **is for caudal orientation in the embryo coils.

Serial sections of three of DiI injected embryos confirmed that the neural crest migrates in large numbers at the tail end region and in smaller numbers at the mid-trunk (Fig. 4). In these almost longitudinal sections, the DiI-positive cells form rostral streams of cells reaching all the way to the dorsal aorta where sympathetic cells will develop (Fig. 4A-D) and the mesonephroi (MN in Fig. 4F). We observed that in snake neural crest cells reach the aorta epithelium as found in chicken [27]. However, when the longitudinal sections were at the mid-trunk region, the migrating cells locate in few numbers between the somites, still following a ventromedial path (Fig. 4E is of a separate embryo, No.4). A transverse section close to the tail end shows robust number of cells migrating along the ventromedial pathway from the neural tube as has been observed for other vertebrates (Fig. 4F, G). This pattern of neural crest streams is very similar to what has been found in for chicken and mice. We also observed epidermis/ectoderm DiI labeling in some embryos, although, we could not determine if it was mislabeling during injection or true DiI-migrating cells that reached those areas in the ectoderm. However, in one embryo we observed cells apparently turning around the dermomyotome as if beginning to enter the area between the ectoderm and mesoderm (Fig. 4H) as has been reported in DiI labeling of Xenopus embryo neural crest [6].

### HNK1 labeling of neural crest

**Figure 5:**
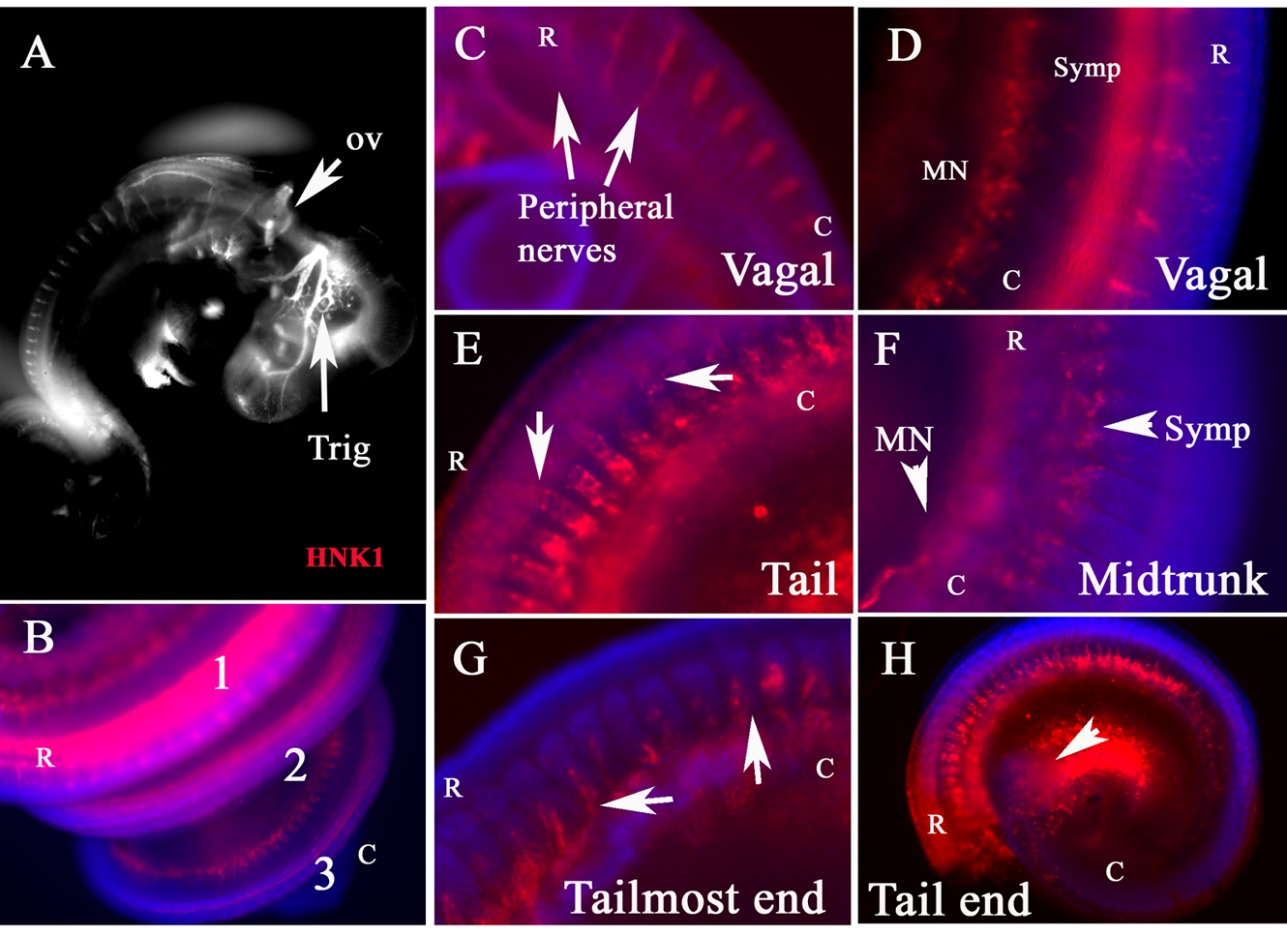
**Wholemount immuno-staining with HNK1**. Snake embryo at st.21 (4POV) was wholemounted with HNK1 antibody (red) and DAPI (blue). **A **and **B **show head and trunk coils respectively. **C **and **D **show sensory ganglia positive for HNK1 at the vagal level. Arrows point spinal nerves positive for HNK1. **E **and **F **show HNK1 cells migrating along ventromedial pathway (arrows in **E**) at tail portion and crest cells at the sympathetic/aorta level or mesonephroi (arrowheads in **F**) at the midtrunk portion. **G **corresponds to the tail-most end, where the first neural crest cells are migrating (arrows). **H **shows the tail end providing a lower magnification of **E **and **G **areas. **MN**: mesonephroi. **Symp**: sympathetic. **R **corresponds to rostral and **C **to caudal.

In order to look in greater detail at migrating neural crest cells in snake embryos, we turned to the HNK1 antibody, known to label neural crest cells in non-mammalian amniotes like birds and crocodilians as well as turtles [17,22,23,28,29]. The wholemount staining pattern of snake embryo neural crest with HNK1 was remarkably similar to the one we observed with live DiI labeling: neural crest cells migrated in large numbers throughout the somite at the most caudal levels (last coil or tail end) and as a small number of cells at the mid-trunk regions (Fig. 5A, B and 5H). Suggesting that these HNK1 labeled cells might well be migrating neural crest cells.

The embryo pictured in Fig. 5 had three coils (st.20) and in each of these regions, it was possible to observe a different stage in the development of the neural crest. In coil 1, the first one posterior to the hindbrain, HNK1 labeled what are likely condensing dorsal root ganglia (DRG, Fig. 5C and 5D). In addition, there is a line of HNK1 positive cells along the same region where the sympathetic chain will form, and at the most ventral portion of the coil, a group of HNK1 positive cells that correspond to where mesonephroi will develop (Fig. 5D). At mid-trunk levels we observed HNK1 labeling by the sympathetic ganglia and mesonephroi areas as well (Fig. 5F). At the tail end of this embryo (still growing) we observed a similar pattern of HNK1 staining as when using DiI (Fig. 5E, G, H): neural crest cells migrating as a rostral stream through the somites towards the mesonephroi region.

**Figure 6:**
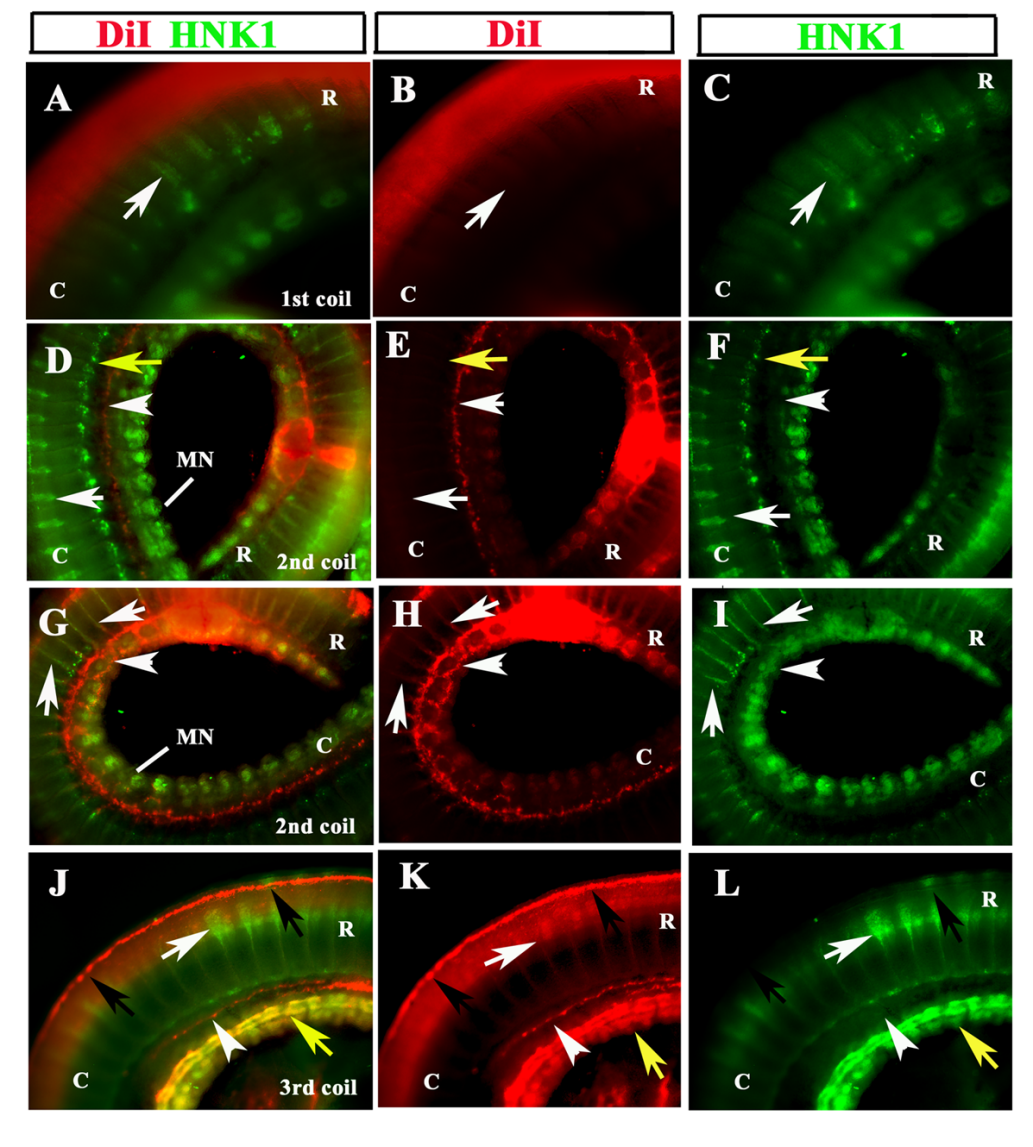
**DiI and HNK1 labels neural crest cells**. DiI (red) labeled st.21 snake embryo No.5 was double stained with HNK1 (green). **A-C **shows 1^st ^coil; **D-I **2^nd ^coil, with **D_F **more rostral versus the **G-I **more caudal portion; and **J-L **the 3^rd ^coil. Each column shows either double channel, or just DiI or HNK1 staining. **A-C **DiI labeling of migrating crest was minimal, HNK1 faintly labeled some cells on the ventral portion of the trunk (white arrow). **D-F **shows strong DiI staining just above (white arrowhead) and in the mesonephroi, the point of injection in this embryo is marked as a large DiI spot. HNK1 staining labeled condensing peripheral ganglia, (white arrow), sympathetic ganglia (yellow arrow) and mesonephroi. **G-H **shows cells along the ventromedial pathway that are positive for both DiI and HNK1 (white arrows). DiI labeling also marked cells that now reached mesonephroi and are populating this tissue around (white arrowhead). **J-L **shows DiI-labeled cells that just delaminated from the neural tube but have not migrated (black arrows) as well as a small group of cells above the mesonephroi (white arrowhead). HNK1 labeled cells moving along the ventromedial pathway (white arrow) as well as the developing mesonephroi (yellow arrow). (The tail-most end of this embryo did not have DiI positive neural tube or migrating cells). **R **is for rostral; **C **is for caudal orientation in the embryo coils.

**Figure 7:**
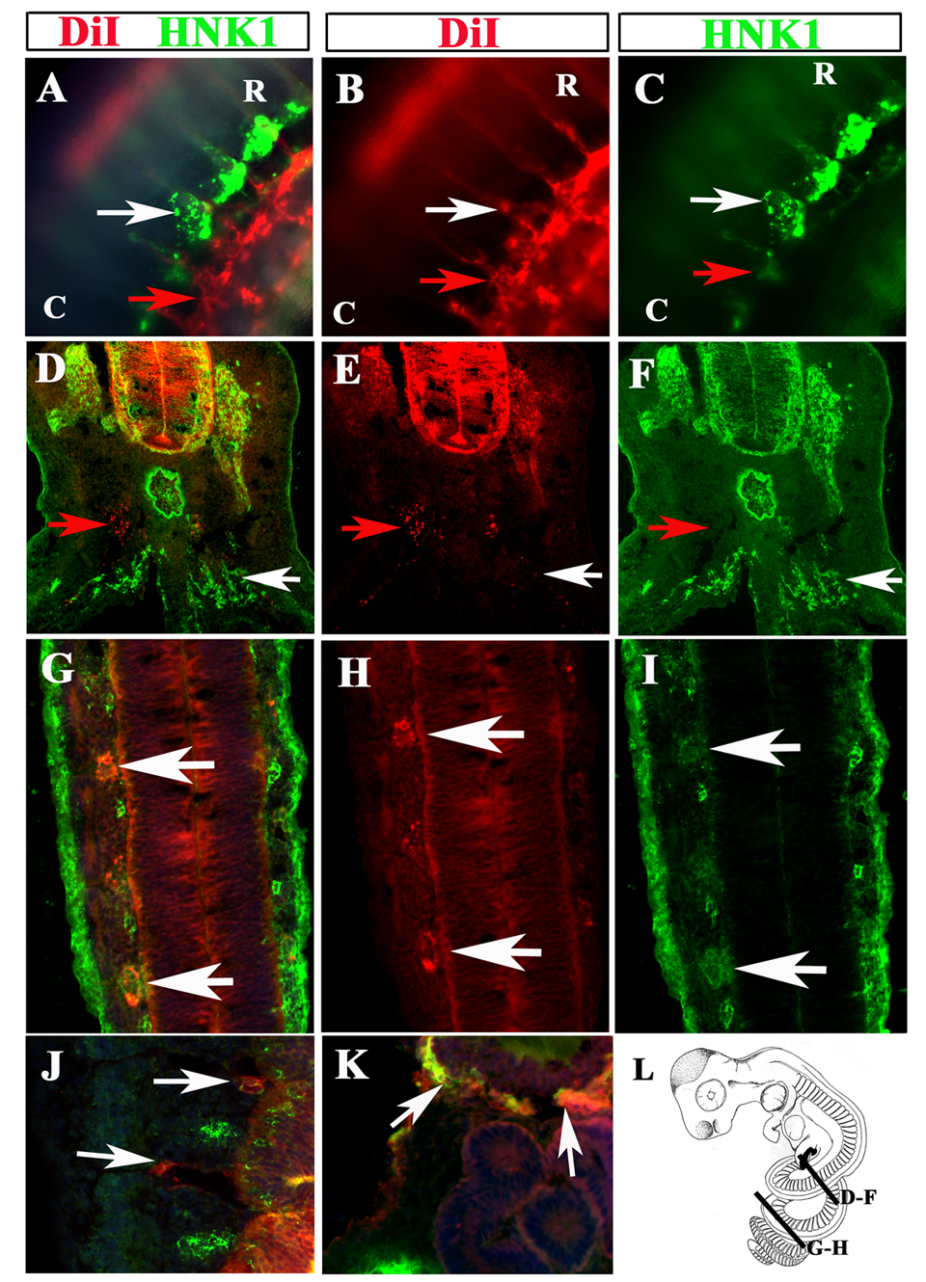
**DiI and HNK1 labels neural crest cells**. DiI (red) and HNK1 (green) st.21 stained snake embryo No.5 sections labels a variety of migrating neural crest. **A-C **show wholemount at the level of second coil, which label DiI migrating cells co-localizing with HNK1 around aorta/ventral portion of trunk (white arrows). There were many DiI cells around the mesonephroi (red arrows). **D-F **show a section on the caudal portion of 1^st ^coil. The only DiI cells were localized in the DRG, which also was strongly stained with HNK1. Other cells were only DiI (red arrows) or only HNK1 (white arrows). **G-I **shows a longitudinal section on the second coil where DiI and HNK1 co-localized, especially in the condensing DRG. Not all DiI were HNK1 positive and vice-versa. **J **shows a higher magnification of one longitudinal section of DiI positive neural crest migrating between the somites (arrows) while HNK1 cells are found in the rostral portion of the somite. **K **corresponds to a section on the 2^nd ^coil showing DiI co-localizing with HNK1 around the mesonephroi/kidneys (re.Fig. 5G-I). **L **is a cartoon depicting the axial levels of the sections shown in this figure. **R **is for rostral; **C **is for caudal orientation in the embryo coils.

When we double labeled one DiI-injected embryo (No.4) with HNK1 we observed one major distinction between DiI and HNK1 labeling alone: strong staining with HNK1 of the mesonephroi, suggesting that HNK1 stains cells in the mesonephroi that are not neural crest cells (Fig. 6). Overlap of HNK1 and DiI in the first coil was not observed (Fig. 6A-C); probably DiI injection in this region was too late to catch delaminating neural crest cells. Similarly, at the rostral portion of the second coil (Fig. 6D-F) HNK1 staining was not only marking what looked like condensing sensory ganglia but also stained very strongly the mesonephroi. Overlap with DiI was minimal in this region, found mostly in the most ventral portion of the DiI stream (see Fig. 7A for details), likely due to late DiI labeling of the neural crest. At the most caudal part of the second coil, double labeling showed robust HNK1 staining in the mesonephroi and strong DiI staining of cells surrounding these structures (arrowhead in Fig. 6G-I) as well as streams of cells positive for HNK1 and DiI along the neural crest pathways (arrows in Fig. 6G). The third coil had very strong double labeling of mesonephroi for DiI and HNK1, while the DiI/HNK1 (yellow arrow in Fig. 6J), double labeled streams along the dorso-ventral pathway was not as robust as in Fig. 6G-I (white arrow in Fig. 6J). DiI however, labeled a population of cells that must have just delaminated from the neural tube as in Fig. 2A but had not time to migrate ventrally (black arrows in Fig. 6J). DiI also continued to mark a stream of cells just above the mesonephroi and beneath another stream of HNK1 cells (arrowhead in Fig. 6J).

A higher magnification of DiI labeled embryo No.5 showed that in some areas (in this instance second coil) there was robust overlap of DiI and HNK1 along the ventromedial pathway (Fig. 7A-C). Interestingly, there were two areas of condensing cells: one strongly labeled with HNK1 (white arrow in Fig. 7A, C) and another strongly labeled with DiI corresponding to the developing mesonephroi (red arrow in Fig. 7A, B). Transverse section at mid-trunk (second coil) level showed that both DiI and HNK1 double-labeled cells in the DRG (Fig. 7D-F). DiI also labeled presumptive sympathetic cells beneath the notochord (red arrow in Fig. 6D-E) and HNK1 cells above/around the aorta probably corresponding to developing mesonephric tubules/adrenal gland (white arrow in Fig. 7D-F). We only observed a small percentage of cells by the dorsal aorta, Longitudinal sections by the third coil showed that HNK1 labeled DiI-positive cells as well as other cells that judging from their segmented pattern could be neural crest (Fig. 7G-I). Interestingly, we observed that the ectoderm was strongly positive for HNK1 (Fig. 7G, I) as has been found for turtles [24]. We also observed in this embryo the inter-somitic path of DiI-positive cells while HNK1-positive neural crest cells were observed migrating through the rostral somite in a classic segmented fashion (Fig. 7J). Finally, at the mesonephroi level we found both DiI and HNK1 positive cells in same locations, as cells surrounding the mesonephroi (Fig. 7K) in this embryo, as well as in the other two that we sectioned.

### Peripheral Nervous system development

**Figure 8:**
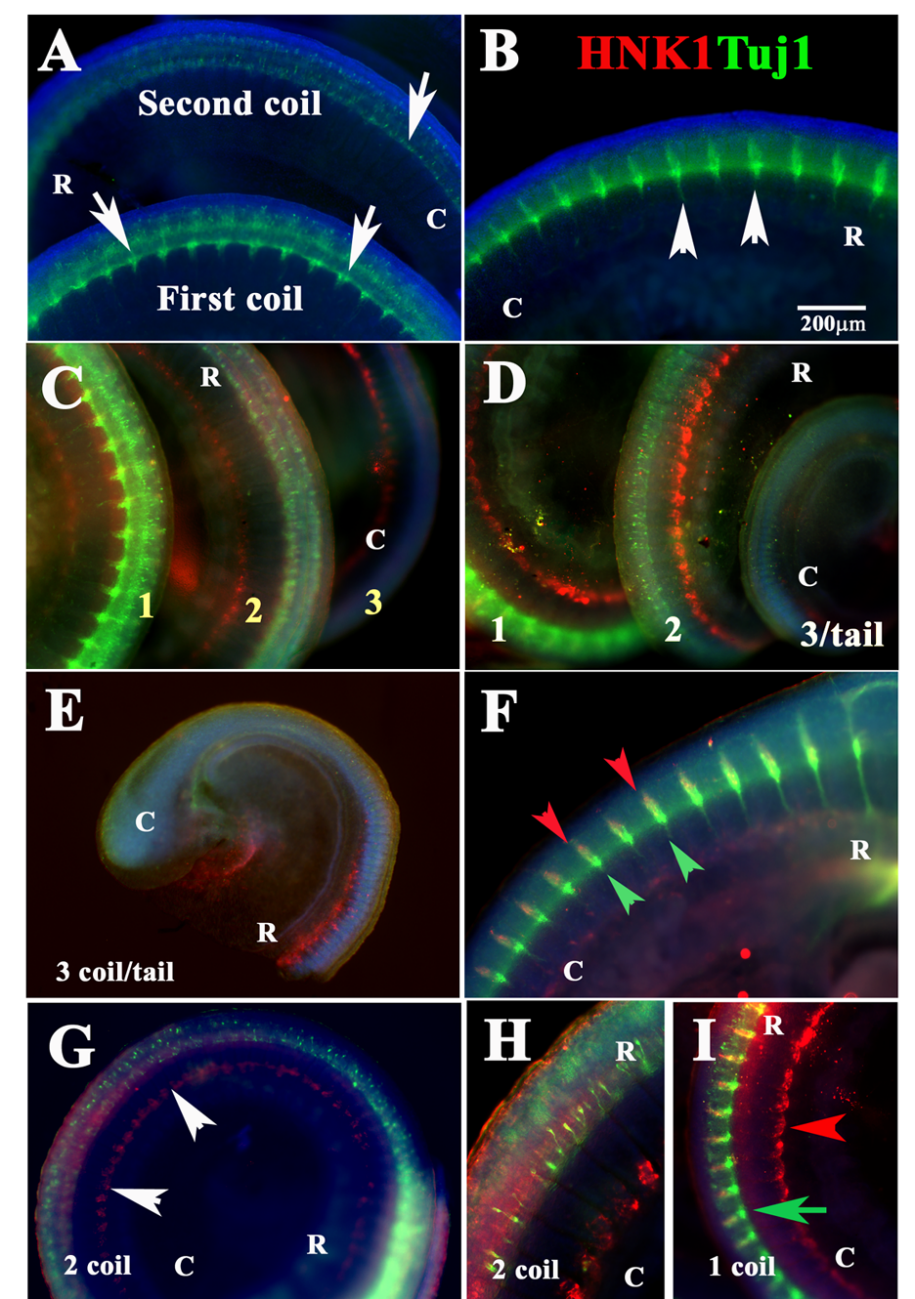
**Peripheral ganglia development in snake embryos**. Wholemount staining of snake embryo with Tuj1 (green in **A **and **B**) or double stained with Tuj1 (green) and HNK1 (red) (**C-I**). DRG development progresses in a rostral to caudal manner: cervical DRGs are clearly mature (arrowheads in **B**) while those in the second coil and tail are not positive for Tuj1 (arrows in **A **and **E**). Only at more rostral levels we observe Tuj1 and HNK1 overlap (red and green arrowheads in **F**). **G **shows second coil where Tuj1 exhibited a spotted pattern, while HNK1 labeled the cells around the aorta as in other figures (arrowheads). **H **shows a higher magnification of **G**. **I **show a higher magnification of first coil. Blue stain corresponds to DAPI labeled nuclei. **R **is for rostral; **C **is for caudal orientation in the embryo coils.

Because peripheral neurons are all neural crest derived, we performed double labeling with the anti-beta-III tubulin neuron-specific antibody, Tuj1, in snake embryo at st.19 (three coils) in order to distinguish between neural crest and differentiating neurons. Wholemount with Tuj1 revealed that the development of dorsal root ganglia (DRG) in snakes progresses in a rostral to caudal manner as in birds and mammals (Fig. 8A-D). We observed that DRGs were strongly labeled with Tuj1 in the first coil at the level of vagal and first trunk somites (Fig. 8C-D); some Tuj1 positive cells in the second coil; and finally (Fig. 8G-H), no Tuj1 signal at the third/tail end coil, although there was HNK1 staining present (Fig. 8D and 8E). Snake DRGs exhibit a different morphology: narrower than what is commonly observed in mouse or chicken. Double labeled embryo for Tuj1 and HNK1 showed that only at the most rostral portions of the embryo do we observe clear overlap for both antibodies (Fig. 8F, I). However, as the trunk progresses caudally after the first coil, the Tuj1 positive cells are inside the neural tube (Fig. 8G and higher magnification in H). Something analogous has been observed for chicken at HH19-21: although HNK1 robustly labels peripheral nervous cells and neural tube as well, Tuj1 I observed in just few of the DRG cells compared with neural tube [30].

**Figure 9:**
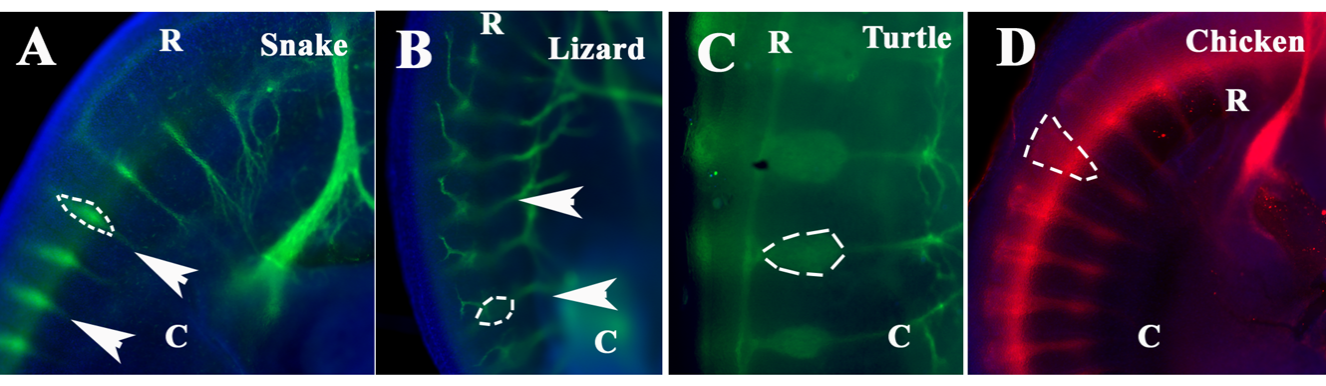
**Peripheral ganglia comparison among archosaurs**. Snake st.21 (**A**), lizard (**B**), turtle (**C**) and chicken HH20 (**D**) and were wholemount stained for Tuj1. Cervical sensory ganglia in snake, lizard and turtle are spindle shaped, while chicken ganglia are triangular (arrowheads). Dotted lines outline the shape of the sensory ganglia. **R **is for rostral; **C **is for caudal orientation in the embryo coils.

In order to highlight possible taxonomic differences/similarities we performed wholemount staining with Tuj1 in lizard (Banded gecko, *Coleonyx variegatus*, Baird, 1859) (Fig. 9B), turtle (Red-eared slider *Trachemys scripta elegans*, Wied-Neuwied, 1839) (Fig. 9C) and chicken (HH21) (Fig. 9D), embryos at somewhat comparable stages of development as much as possible, since these organisms differ in their embryogenesis and it was difficult to obtain the proper samples. The stages of development were determined by the development of branchial arches, nasal pit and the eye. Comparison between these four phylogenetically related organisms highlighted that snake peripheral ganglia have different shape from birds and mammals. We found that the shape of peripheral ganglia in these three reptilian species was more similar among themselves than to birds (Fig. 9D) or rodents (data not shown). We found that the reptilian peripheral ganglia had an elongated fusiform shape (Fig. 9A-C), rather than the inverted triangles typically observed in birds and mammals (Fig. 9D and data not shown for mouse). From all these species, snake showed the smallest ganglia compared with a lizard or turtle (Fig. 9A).

## Discussion

In the present study we examined the migration of snake trunk neural crest during development by using live cell labeling and neural crest markers. We found that neural crest cells in the king snake (*Lampropeltis getula California*) follow what seem to be highly conserved migratory patterns among amniotes as shown by HNK1 staining in turtles and birds [17,24].

It is known that neural crest cells move rapidly, and within 8 hrs after delamination from the neural tube they have reached their destinations in avian and mouse embryos [31,32]. In our study we incubated the embryos after labeling the neural tube with DiI for 12 and 24 hrs, and found that after this period of time, large number of cells has reached the same locations as in other vertebrates including the dorsal aorta, mesonephros, and developing gut. We did not observe a large number of DiI neural crest cells in the developing gut, presumably because the labeling was done too late to mark the delaminating vagal neural crest [33].

Neural crest migration has been studied by using HNK1 in non-avian reptiles, including crocodile and turtle [23,24]. Hou and Takeuchi found that the migratory pattern of cranial and trunk neural crest in turtles followed that of birds [34]. In our present study we found that snake trunk neural crest followed the essentially same migratory pathways as described for turtles and and other non-avian reptiles [24,35,36] suggesting conservation of these core pathways among all reptiles and amniotes.

Despite conservation of the main trunk neural crest migratory pathways, we did observe some differences in how these pathways were utilized. Snakes have the ability to grow their tail extensively (up to 350 somites length) allowing the simultaneous observation of the newly delaminated neural crest cells at the tail end [25] as well as the beginning of differentiating neural crest in the first coils. Our DiI labeling in a colubridae snake showed cell streams in what we term a 'dual pathway' of migration. The *first *group of delaminating neural crest cells followed very much the migratory pathways observed in avian and mammalian embryos; cells avoiding the caudal portion of the somites and moving along the ventromedial path towards the dorsal aorta region [5,37]. However, at more rostral axial levels, a *second *large group of late-migrating trunk neural crest cells was observed moving as narrow streams between the somites. Such intersomitic migration has been observed for chicken as an early event in the first delaminating cells [5,38], but not in late migrating neural crest cells. Interestingly, no intersomitic migration has been observed in non-avian reptiles [24]. These findings suggest heavy utilization of the intersomitic pathway by late-migrating neural crest cells may be a derived feature of snakes, or an aspect of development lost in birds and mammals. More detailed studies of trunk neural crest migration in other non-avian reptiles will be needed to determine which scenario is more likely.

Recently, it was shown that mice neural crest cells will migrate along the intersomitic space when both Sema3A, F and their receptors (Neuropilin 1 and 2) are absent [39]. This finding reinforces the importance of such inhibitory molecules (somite environment) in controlling neural crest migration and suggests they may play important roles in altering migratory patterns during evolution.

In addition to differences in the migration of late-emerging neural crest cells, between snake and other amniotes, HNK1 staining revealing a difference in the shape of dorsal root ganglia (DRGs) between non-avian reptiles, mouse, and chick. Whereas mouse and chick DRGs are triangular, those of snake, gecko and turtle are smaller and spindle-shaped. This likely reflects some level of convergence between the structure of bird and mammalian DRGs related to the evolution of a more active lifestyle in these two warm-blooded groups. Such convergence has been reported in evolution of the bird and mammalian hearts [40].

Another unexpected result from this study was the large proportion of trunk neural crest cells found in the mesonephric region by using DiI and HNK1 labeling. We found a considerable number of neural crest cells that migrated beyond the sympathetic ganglia region towards the mesonephroi. These cells do not correspond to the ones reported previously around the aorta [5]. The mesonephroi in birds are derived from mesodermal tissues. The adrenal cortex primordia in snakes align as a strand between the mesonephroi and the dorsal aorta, although later it will lose contact with the kidneys [41]. It is assumed that snake neural crest cells migrate towards the mesonephroi region and give rise to the chromaffin adrenal cells and even that snake mesonephroi could be partly crest derived [41,42]. The present study supports a crest derived origin for the chromaffin cells, though not so for mesonephroi itself. Our results show DiI and HNK1 labeled cells around developing tubules, not as part of the tubules themselves. Therefore, snake trunk neural crest likely gives rise to the adrenal chromaffin cells, although we could not determine the ultimate fate of all those cells migrating towards the developing kidney. Future studies looking more closely, and after longer labeling period, will address this issue.

## Conclusions

In summary, our study is the first description of trunk neural crest cell migration in snakes, using both vital dye labeling, and immunofluorescence. The pattern of migration in this organism initially follows the rostral ventromedial pathway typical of model amniotes and then switches to a late inter-somitic pathway. This late dependence on the intersomitic pathway appears may be unique to snakes, or may have been lost in other amniotes. Our results highlight both conserved and divergent features of snake trunk neural crest migration.

## Methods

### Collection and Staging of Embryos

Eggs of *Lampropeltis getula californiae *(Blainville, 1835), the common California king snake, were gathered from the herpetarium collection at CSUN two to three days after oviposition and embryos were collected within the next three days. Animal use and up keeping was according to approved protocols by the IACUC board of CSUN (Protocol #0506-012c). The embryos were staged according to Zehr's normal table [26].

Embryos were removed from egg cases by incision on one of the top edges (embryos are usually located in the midsection of the soft eggshell), the contents were emptied onto a Petri dish and after locating the embryo the shell was fully opened. Embryos were fixed in Carnoy's (70% ethanol, 20% formaldehyde and 10% glacial acetic acid) overnight for 24 hrs at 4°C, and then stored in 100% methanol at -20°C until histology preparation. Embryos went through prolonged (several hours each) dehydration steps in alcohol series and then placed in histosol for clearing. The tissues were then immersed in hot paraffin (McCormick Scientific Paraplast Plus) in a vacuum oven for two days before preparing the blocks and sectioning. Embryos were sectioned (10-12 μm) with a microtome, placed on Super-Frost slides and dried overnight at 37°C on a slide warmer.

Lizard embryos (Banded gecko, *Coleonyx variegatus*, Baird, 1859) were collected from three eggs from the herpetarium collection at CSUN after oviposition. Turtle embryos (Red-eared slider *Trachemys scripta elegans*, Wied-Neuwied, 1839) were collected from a freshly laid clutch in a California pond and embryos were fixed two weeks after oviposition. Chicken embryos were obtained from a Los Angeles purveyor of fertilized farm after incubating them to HH17 [43].

### Immunohistochemistry

Snake tissue sections were re-hydrated in histosol and a graded series of ethanol washes (histosol, 100, 90, 70, 50 and 25% ethanol washes in water) and then equilibrated in PBS (Dulbecco's) before blocking in PBS containing 10% expired FBS and 1% Triton X-100 for 12 hrs. Primary antibodies were added in a 1:100 (or 1:1 for hybridoma supernatants) dilution in PBS and slides were incubated for two days at 4°C. After washing the sections in PBS for at least 20 minutes, secondary antibodies (Alexa fluoroprobes conjugated to anti-rabbit or anti-mouse IgG, Invitrogen) were added for 30 min and washed in PBS for immuno-fluorescence visualization and cover-slipped with Permount. Pictures of sections were taken using Axiovision LE software (Zeiss™) with an AxioCam black and white camera attached to a Zeiss AxioimagerA1 upright fluorescent microscope and assembled into figures using Adobe Photoshop 7. Primary antibodies were: HNK1 hybridoma collected at Caltech from a supernatant prepared following ATCC (Cat. No. TIB-200) instructions for HNK1, Tuj1 from Sigma and GFP antibody from Molecular Probes (Invitrogen).

### Wholemount Immunofluorescence

Embryos were blocked overnight in blocking buffer (Phosphate Buffered Saline (PBS) containing 10% expired FBS and 1% Triton X-100 for 12 hrs, and then incubated with primary antibodies in PBS overnight at 4°C. The next day, embryos were extensively washed with PBS and incubated with secondary antibodies (anti-mouse or anti-rabbit-Alexa 488/594, Invitrogen, Molecular Probes). The following day the embryos were washed extensively for at least 4 hrs in PBS and photographed with either a Zeiss A-1 AxioImager or a LUMAR.

### DiI labeling

We tested two strategies at the beginning of these experiments: in the first, three snake embryos at st.18-19 [26] were collected and while still alive injected with DiI (cell tracker CM-DiI, C-7001, Invitrogen/Molecular Probes) (diluted 1:10 in ethanol in 10% sucrose) inside the neural tube along its length and hindbrain regions (Additional file 2). The first embryo was then placed on a Petri dish after rinsing in Ringer solution and incubated with 5 ml of DMEM, 10% FBS, penicillin and streptomycin at 37°C for 24 hrs. The second and third embryos were cultured in 3 ml of a 1:1:1 mix of: a) DMEM/10%FBS, b) equal amounts of snake egg white and yolk, and c) 100% FBS at 25°C and 37°C for 12 hrs. In the second strategy, we cut the embryos leaving as much as possible its surrounding membranes and cultured them in DMEM, 20% FBS, penicillin and streptomycin at 25°C for 24 hrs. All embryos survived after DiI injection. At the end of incubation, the surviving cultured embryos were fixed for 1 hr to keep morphology, then placed in a vial for overnight fixing at 4°C and kept there until analysis. In both strategies we injected DiI with a mouth pipette after the hindbrain blowing gently to fill the neural tube all the way until the tail. In embryos that were st.20 we needed to inject around the beginning of second coil to make the DiI reach until the tail, thus these embryos had two points of injection (see Additional file 2).

## Abbreviations

**HNK1**: human natural killer-1 cell antibody, obtained from ATCC cell culture (Cat. No. TIB-200); **DiI**: 3H-Indolium, 5-[[4-(chloromethyl)benzoyl]amino]methyl]-2-[3-(1,3-dihydro-3,3-dimethyl-1-octadecyl-2H-indol-2-ylidene)-1-propenyl]-3,3-dimethyl-1-octadecyl-, chloride; **GFP**: green fluorescent protein; **DRG**: dorsal root ganglion; **PBS**: phosphate buffer saline, Dulbecco's recipe; **DMEM**: Dulbecco minimal essential medium; **FBS**: fetal bovine serum; **POP**: post-oviposition.

## Authors' contributions

All the authors read and approved the manuscript. MR (undergraduate student): performed DiI injections, immunostaining. KZ (high school student): performed immunostaining of snake embryos. ID (undergraduate student): snake cartoon. MEdB: PI, immunofluorescence pictures, manuscript writing.

## Supplementary Material

Additional file 1 **DiI injection of Snake embryos**. Table describes the incubation conditions for each of the 5 DiI injected snake embryos described in this paper. Click here for file

Additional file 2 **DiI injection sites in Snake embryos**. Snake cartoon of stages 19 and 21 indicating with arrows the entry point of DiI injection between the two neural tube folds. Click here for file

## References

[B1] Hall BK The Neural Crest and Neural Crest Cells in Vertebrate Development and Evolution 2009 New York: Springer-Verlag

[B2] Baker CV Neural Crest and Cranial Ectodermal Placodes 2005 4 New York: Springer, New York

[B3] Freitas R Zhang G Albert JS Evans DH Cohn MJ Developmental origin of shark electrosensory organs Evol Dev 2006 8 74 80 10.1111/j.1525-142X.2006.05076.x 16409384

[B4] Smith M Hickman A Amanzee D Lumsden A Thorogood P Trunk neural crest origin of caudal fin mesenchyme in the zebrafish *Danio rerio* Proc R Soc Lond B Biol Sci 1994 256 137 145 10.1098/rspb.1994.0061

[B5] Serbedzija GN Bronner-Fraser M Fraser SE A vital dye analysis of the timing and pathways of avian trunk neural crest cell migration Development 1989 106 809 16 2562671 10.1242/dev.106.4.809

[B6] Collazo A Bronner-Fraser M Fraser SE Vital dye labelling of Xenopus laevis trunk neural crest reveals multipotency and novel pathways of migration Development 1993 118 363 76 7693414 10.1242/dev.118.2.363

[B7] Eisen JS Weston JA Development of the neural crest in the zebrafish Dev Biol 1993 159 50 9 10.1006/dbio.1993.1220 8365574

[B8] Sadaghiani B Vielkind JR Distribution and migration pathways of HNK-1-immunoreactive neural crest cells in teleost fish embryos Development 1990 110 197 209 1706978 10.1242/dev.110.1.197

[B9] Sauka-Spengler T Bronner-Fraser M Evolution of the neural crest viewed from a gene regulatory perspective Genesis 2008 46 673 682 10.1002/dvg.20436 19003930

[B10] Sauka-Spengler T Bronner-Fraser M Insights from a sea lamprey into the evolution of neural crest gene regulatory network Biol Bull 2008 214 303 14 10.2307/25470671 18574106

[B11] Horigome N Myojin M Ueki T Hirano S Aizawa S Kuratani S Development of cephalic neural crest cells in embryos of Lampetra japonica, with special reference to the evolution of the jaw Dev Biol 1999 207 287 308 10.1006/dbio.1998.9175 10068464

[B12] Aoki Y Saint-Germain N Gyda M Magner-Fink E Lee YH Credidio C Saint-Jeannet JP Sox10 regulates the development of neural crest-derived melanocytes in Xenopus Dev Biol 2003 259 19 33 10.1016/S0012-1606(03)00161-1 12812785

[B13] Kulesa P Ellies DL Trainor PA Comparative analysis of neural crest cell death, migration, and function during vertebrate embryogenesis Dev Dyn 2004 229 14 29 10.1002/dvdy.10485 14699574

[B14] Nakao T Ishizawa A Development of the spinal nerves in the lamprey: III. Spinal ganglia and dorsal roots in 26-day (13 mm) larvae J Comp Neurol 1987 256 369 85 10.1002/cne.902560306 3571511

[B15] Teillet MA Kalcheim C Le Douarin NM Formation of the dorsal root ganglia in the avian embryo: segmental origin and migratory behavior of neural crest progenitor cells Dev Biol 1987 120 329 47 10.1016/0012-1606(87)90236-3 3549390

[B16] Serbedzija GN Fraser SE Bronner-Fraser M Pathways of trunk neural crest cell migration in the mouse embryo as revealed by vital dye labelling Development 1990 108 605 12 2387238 10.1242/dev.108.4.605

[B17] Bronner-Fraser M Analysis of the early stages of trunk neural crest migration in avian embryos using monoclonal antibody HNK-1 Dev Biol 1986 115 44 55 10.1016/0012-1606(86)90226-5 3516760

[B18] Shedlock AM Botka CW Zhao S Shetty J Zhang T Liu JS Deschavanne PJ Edwards SV Phylogenomics of nonavian reptiles and the structure of the ancestral amniote genome Natl Acad ProcSci USA 2007 104 2767 72 10.1073/pnas.0606204104 PMC1815256 17307883

[B19] Meyer A Zardoya R Recent advances in the (molecular) phylogeny of vertebrates Annual Review of Ecology Evolution and Systematics 2003 34 311 338 10.1146/annurev.ecolsys.34.011802.132351

[B20] Rest JS Ast JC Austin CC Waddell PJ Tibbetts EA Hay JM Mindell DP Molecular systematics of primary reptilian lineages and the tuatara mitochondrial genome Molecular Phylogenetics and Evolution 2003 29 289 297 10.1016/S1055-7903(03)00108-8 13678684

[B21] Kundrat M Heterochronic shift between early organogenesis and migration of cephalic neural crest cells in two divergent evolutionary phenotypes of archosaurs: crocodile and ostrich Evolution & Development 2009 11 535 546 10.1111/j.1525-142X.2009.00352.x 19754710

[B22] Clark K Bender G Murray BP Panfilio K Cook S Davis R Murnen K Tuan RS Gilbert SF Evidence for the neural crest origin of turtle plastron bones Genesis 2001 31 111 7 10.1002/gene.10012 11747201

[B23] Kundrat M HNK-1 immunoreactivity during early morphogenesis of the head region in a nonmodel vertebrate, crocodile embryo Naturwissenschaften 2008 95 1063 72 10.1007/s00114-008-0426-4 18668221

[B24] Hou L Takeuchi T Neural crest development in reptilian embryos, studied with monoclonal antibody, HNK-1 Zoolog Sci 1994 11 423 431

[B25] Gomez C Ozbudak EM Wunderlich J Baumann D Lewis J Pourquie O Control of segment number in vertebrate embryos Nature 2008 454 335 9 10.1038/nature07020 18563087

[B26] Zehr DR Stages in the Normal Development of the Common Garter Snake, Thamnophis sirtalis sirtalis Copeia 1962 1962 322 329 10.2307/1440898

[B27] Thiery JP Duband JL Delouvee A Pathways and mechanisms of avian trunk neural crest cell migration and localization Dev Biol 1982 93 324 43 10.1016/0012-1606(82)90121-X 7141101

[B28] Tucker GC Aoyama H Lipinski M Tursz T Thiery JP Identical reactivity of monoclonal antibodies HNK-1 and NC-1: conservation in vertebrates on cells derived from the neural primordium and on some leukocytes Cell Differ 1984 14 223 30 10.1016/0045-6039(84)90049-6 6207939

[B29] Kalcheim C Le Douarin NM Requirement of a neural tube signal for the differentiation of neural crest cells into dorsal root ganglia Dev Biol 1986 116 451 66 10.1016/0012-1606(86)90146-6 3525281

[B30] De Bellard ME Barembaum M Arman O Bronner-Fraser M Lunatic fringe causes expansion and increased neurogenesis of trunk neural tube and neural crest populations Neuron Glia Biol 2007 3 93 103 18414598 10.1017/S1740925X07000683 PMC2293300

[B31] Kasemeier-Kulesa JC Kulesa PM Lefcort F Imaging neural crest cell dynamics during formation of dorsal root ganglia and sympathetic ganglia Development 2005 132 235 45 10.1242/dev.01553 15590743

[B32] Kulesa PM Fraser SE In ovo time-lapse analysis of chick hindbrain neural crest cell migration shows cell interactions during migration to the branchial arches Development 2000 127 1161 72 10683170 10.1242/dev.127.6.1161

[B33] Le Douarin NM Teillet MA The migration of neural crest cells to the wall of the digestive tract in avian embryo J Embryol Exp Morphol 1973 30 31 48 4729950

[B34] Sadaghiani B Crawford BJ Vielkind JR Generation of poly- and mono-clonal antibodies against trout fibronectin (FN) and their use in FN immunodetection in teleost fishes Biochem Cell Biol 1994 72 343 8 10.1139/o94-047 7893474

[B35] Dupin E Ziller C Le Douarin NM The avian embryo as a model in developmental studies: chimeras and in vitro clonal analysis Curr Top Dev Biol 1998 36 1 35 10.1016/S0070-2153(08)60493-7 9342519

[B36] Trainor PA Specification of neural crest cell formation and migration in mouse embryos Semin Cell Dev Biol 2005 16 683 93 10.1016/j.semcdb.2005.06.007 16043371

[B37] Bronner-Fraser M Neural crest cell formation and migration in the developing embryo Faseb J 1994 8 699 706 8050668 10.1096/fasebj.8.10.8050668

[B38] Serbedzija GN Bronner-Fraser M Fraser SE Vital dye analysis of cranial neural crest cell migration in the mouse embryo Development 1992 116 297 307 1283734 10.1242/dev.116.2.297

[B39] Schwarz Q Maden CH Davidson K Ruhrberg C Neuropilin-mediated neural crest cell guidance is essential to organise sensory neurons into segmented dorsal root ganglia Development 2009 136 1785 1789 10.1242/dev.034322 19386662 PMC2680105

[B40] Koshiba-Takeuchi K Mori AD Kaynak BL Cebra-Thomas J Sukonnik T Georges RO Latham S Beck L Henkelman RM Black BL Reptilian heart development and the molecular basis of cardiac chamber evolution Nature 2009 461 95 8 10.1038/nature08324 19727199 PMC2753965

[B41] Rupik W Early development of the adrenal glands in the grass snake Natrix natrix L. (Lepidosauria, Serpentes) Adv Anat Embryol Cell Biol 2002 164 I XI 1-102. 12080925 10.1007/978-3-642-55977-8

[B42] Gabe M [Histologic data on the endocrine pancreas of Lepidosaurians (reptiles)] Ergeb Anat Entwicklungsgesch 1970 42 3 61 4191848

[B43] Hamburger V Hamilton HL A series of normal stages in the development of the chicken embryo J Morph 1951 88 49 52 10.1002/jmor.1050880104 24539719

