# Supplementary material for: Emergence and migration of trunk neural crest cells in a snake, the California Kingsnake (Lampropeltis getula californiae)
Source: BMC Dev Biol. 2010 May 18;10:52. doi: 10.1186/1471-213X-10-52 (PMC2886003; doi:10.1186/1471-213X-10-52)
Supplement: Additional file 1 — DiI injection of Snake embryos. Table describes the incubation conditions for each of the 5 DiI injected snake embryos described in this paper. [file 1471-213X-10-52-S1.DOC]

|  | **Embryo No.1** | **Embryo No.2** | **Embryo No.3** | **Embryo No.4** | **Embryo No.5** |
| --- | --- | --- | --- | --- | --- |
| **Culture conditions** | Yolk+DMEM+20%FBS | Yolk+DMEM+20%FBS | Vitelline membranes +DMEM+20%FBS | Vitelline membranes +DMEM+20%FBS | Vitelline membranes +DMEM+20%FBS |
| **Incubation period** | 12hrs | 12hrs | 24hrs | 24hrs | 24hrs |
| **Stage of injection** | St.19 | St.19 | St.20 | St.20 | St.21 |
| **DiI-positive coils** | 1-2 | 1-2 | 2 | 1-3 | 1-3 ¾ |
| **DiI migrated NCC** | Very few | Yes | Very few | Extensive | Extensive |
| **Shown in Figure** | 2 | 2, 3, 4 | X | 2, 6, 7 | 2, 7 |
| **Temperatures** | 37^o^C | 25^o^C | 25^o^C | 25^o^C | 25^o^C |
