# Supplementary figures and images for: Emergence and migration of trunk neural crest cells in a snake, the California Kingsnake (Lampropeltis getula californiae)
Source: BMC Dev Biol. 2010 May 18;10:52. doi: 10.1186/1471-213X-10-52 (PMC2886003; doi:10.1186/1471-213X-10-52)

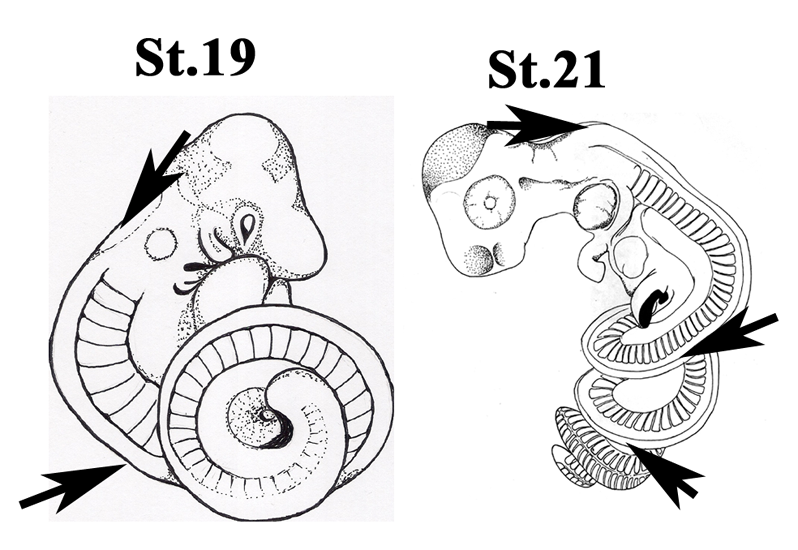

Supplement: Additional file 2 — DiI injection sites in Snake embryos. Snake cartoon of stages 19 and 21 indicating with arrows the entry point of DiI injection between the two neural tube folds. [file 1471-213X-10-52-S2.TIFF]
